# Education Research: Junior Neurology Residents Achieve Competency but Not Mastery After a Brief Acute Ischemic Stroke Simulation Course

**DOI:** 10.1212/NE9.0000000000200071

**Published:** 2023-05-26

**Authors:** Catherine S.W. Albin, Melissa B. Pergakis, Erika J. Sigman, Nirav R. Bhatt, Spencer K. Hutto, Sitara Koneru, Ehizele M. Osehobo, Joaquin A. Vizcarra, Nicholas A. Morris

**Affiliations:** From the Department of Neurology and Neurosurgery (C.S.W.A., E.J.S.), Emory University School of Medicine, Atlanta, GA; Department of Neurology (M.B.P., N.A.M.), Program in Trauma, University of Maryland School of Medicine, Baltimore; Department of Neurology (N.R.B.), University of Pittsburgh School of Medicine, PA; and Department of Neurology (S.K.H., S.K., E.M.O., J.A.V.), Emory University School of Medicine, Atlanta, GA.

## Abstract

**Background and Objectives:**

Simulation bootcamps are used to onboard neurology trainees. It is not known whether these bootcamps result in competency for acute ischemic stroke (AIS).

**Methods:**

For this prospective, single-center pre-post educational intervention study, the Angoff standard setting method was used to determine a Minimum Passing Score (MPS) and Mastery Score for 2 AIS simulations. Junior neurology residents completed precourse knowledge and confidence assessments and had traditional didactic teaching. A week later, each resident completed the first scored AIS simulation. Each resident then practiced stroke care in an unscored simulation. Two to 8 weeks later, each resident was evaluated in an unannounced AIS simulation (the post-test). Postgraduate year (PGY)-3 adult neurology senior residents also completed a knowledge and confidence assessment and were scored on just the AIS post-test case. Using independent and paired *t* tests, respectively, we compared the junior residents' retention test performance to their baseline assessment and to senior residents' performance.

**Results:**

Thirteen junior residents (9 PGY-2 adult neurology residents and 4 PGY-3 child neurology residents) participated in the course. Only 3 junior residents (23%) initially achieved the MPS in the first AIS simulation. After the simulation course, 9 junior residents (69%) achieved the MPS threshold. Although none achieved mastery, junior residents' mean performance score in the simulation improved (mean score preintervention [SD] = 10.3 [2.8] vs mean score postintervention [SD] = 15.7 [2.6], *p* < 0.001) and their confidence increased (mean score preintervention [SD] = 3.3 [1.9] vs mean score postintervention [SD] = 4.9 [1.2], *p* < 0.001, *d* = 1.7). Eight PGY-3 adult neurology residents were scored on the AIS post-test. Five reached MPS (63%), and 1 demonstrated mastery. The simulation scores of the postcourse juniors and seniors were similar (junior resident mean score [SD] = 15.7 [2.6] vs senior resident mean score [SD] = 16.0 [2.5], *p* = 0.793).

**Discussion:**

A brief AIS simulation course may improve junior residents' performance and confidence to a level comparable with senior residents, although not to mastery.

Acute ischemic stroke (AIS) is a leading cause of disability and death worldwide.^1^ With ongoing advances in acute reperfusion therapy, it is critical that these patients are managed expediently as every 1-hour treatment delay results in less functional independence.^2^ Day-long sessions in simulation laboratory results in which residents have intensive practice in the care of neurologic emergencies and procedural training—“simulation bootcamps”—have been used in neurology as an engaging way to improve knowledge in the management of acute neurologic conditions, such as AIS.^3–8^ These simulation bootcamps are highly rated by trainees and result in improved knowledge and self-reported confidence.^3–6^ Two independent academic medical centers have also shown that integration of simulation-based training was associated with improved stroke quality metrics.^7,8^ However, while the authors of both studies hypothesized that resident training was the factor that improved these metrics, neither study directly assessed resident performance in follow-up. Thus, it is uncertain how simulation-based training bootcamps in AIS management produce a behavior change among participating residents.

Simulation-based mastery learning (SBML) is a form of competency-based learning where learners are required to meet a very high level of skill before the completion of training.^9^ A curriculum combining didactics and SBML has previously been used to promote durable competency in the recognition and management of status epilepticus (SE).^10^ The SE curriculum was very effective in achieving an enduring and measurable competency: all residents achieved the consensus-defined Minimum Passing Score (MPS) on the post-test, which was durably retained in a follow-up in situ simulation. However, the average time from pretest to post-test was 71.25 days, as nearly half of the resident's required additional practice. Although effective in demonstrating improved resident performance, this method is resource and time intensive.

We thus sought to study the degree to which a consensus-defined MPS could be achieved by junior residents who participated in a less time-intensive intervention: a didactic session followed by a 1-day stroke onboarding simulation course in which they received directed feedback and time for deliberate practice of AIS management. As we did not require residents to achieve a MPS before the unannounced follow-up scored simulation, we compared their follow-up performance with that of senior residents in the same case. We hypothesized that the stroke onboarding course would (1) promote junior residents' achievement of at least minimal competency in AIS management and (2) accelerate junior residents' performance to that of senior residents.

## Methods

### Setting and Study Design

This prospective, single-center pre-post educational intervention study was performed from July through September 2022. Residents were assured that their performance would not affect their grades and that all scores would be anonymously collected through Qualtrics using a secured alpha numeric code known only to the lead author (C.S.W.A.). Participation in the simulation curriculum was required; however, participation in the research component was optional. Junior participants were residents in their first year of neurology training (9 postgraduate year [PGY]-2 neurology residents and 4 PGY-3 child neurology residents). Senior resident comparators were PGY-3 adult neurology residents who had completed a year of being first responders to acute stroke while on their stroke rotations and had attended multiple stroke didactics throughout the prior year. A sample size calculation was not made, as we planned to enroll as many residents at our institution who were willing to participate.

### Protocol

Before completing their intern year, all junior participants were asked to complete a questionnaire regarding their prior experience with neurologic emergencies including acute stroke (Table 1). Each participant also completed a multiple-choice knowledge test about AIS and confidence self-assessment (eAppendix 1, links.lww.com/NE9/A26). Participants then rated their confidence in the management of 12 AIS subcompetencies and 11 Emergency Neurological Life Support (ENLS) topics.^11^

**Table 1:**
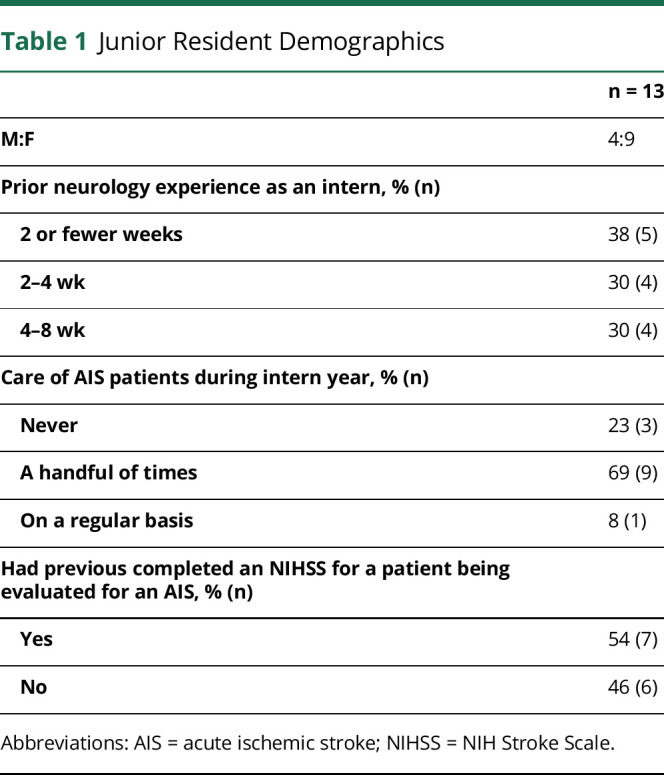
Junior Resident Demographics

	n = 13
M:F	4:9
Prior neurology experience as an intern, % (n)	
2 or fewer weeks	38 (5)
2–4 wk	30 (4)
4–8 wk	30 (4)
Care of AIS patients during intern year, % (n)	
Never	23 (3)
A handful of times	69 (9)
On a regular basis	8 (1)
Had previous completed an NIHSS for a patient being evaluated for an AIS, % (n)	
Yes	54 (7)
No	46 (6)

Abbreviations: AIS = acute ischemic stroke; NIHSS = NIH Stroke Scale.

**Figure 1:**
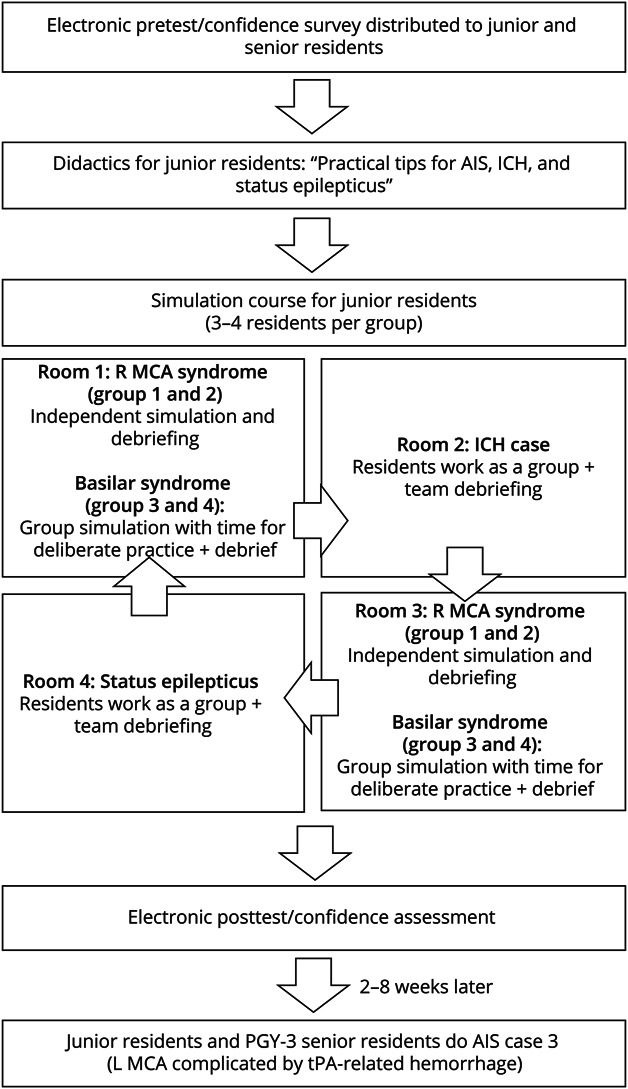
Curriculum and Study Design A schematic representation of the curriculum process. AIS = acute ischemic stroke; ICH = intracerebral hemorrhage; MCA = middle cerebral artery; PGY = postgraduate year; tPA = tissue plasminogen activator.

On the first day of the new academic year, all junior residents attended a didactic session on the management of AIS, SE, and intracerebral hemorrhage (ICH). After the didactic sessions, participants completed the same multiple-choice knowledge test. A week after the didactic session, all trainees participated in a neurologic emergencies simulation bootcamp, rotating through 4 scenarios (Figure 1). Two of the rooms portrayed AIS. Both rooms simulated a middle cerebral artery (MCA) syndrome case (baseline competency assessment) until all residents had been independently scored. After a break, both acute stroke rooms simulated the scenario of coma due to acute basilar artery thrombosis. The other 2 rooms simulated ICH and SE cases that were not scored. Trainees completed the MCA syndrome case independently while all other scenarios were completed as a team.

During all the cases, residents were encouraged to use cognitive aids. The NIH Stroke Scale (NIHSS) scorecard and tissue plasminogen activator (tPA) exclusion criteria were provided. After each case, residents received tailored Debriefing with Good Judgment,^12^ which encourages uncovering the underlying frames for decision and self-reflection. Debriefing was performed one-on-one with one of the course faculty for the first AIS case. In cases where residents participated in teams, they received debriefing as a team. After completion of the simulation course, residents completed the multiple-choice knowledge test (eAppendix 4, links.lww.com/NE9/A29) for the third time and repeated the confidence self-assessment. Once all knowledge and confidence scores were received, they were eligible to receive a $10 gift card.

### Simulation Case Development

Each simulation case was developed based on American Heart Association/American Stroke Association Stroke Guidelines^13,14^ and Neurocritical Care Society's Guidelines for Status Epilepticus.^15^ To improve proficiency in AIS, we designed 2 AIS cases and used a previously validated AIS case for the follow-up assessment.^16^ The baseline assessment (AIS case 1, eAppendix 2, links.lww.com/NE9/A27) featured a patient who was suffering from an acute right MCA syndrome and presented within the thrombolytic window. The case was created by 1 board-certified neurointensivist (C.S.W.A.), reviewed by a board-certified neurointensivist at a different facility (N.A.M.), and a board-certified vascular neurologist (N.R.B.). The final checklist was a consensus among all 3 faculties. The basilar artery thrombosis case (AIS case 2, eAppendix 3, links.lww.com/NE9/A28) was developed in a similar fashion. The post-test case (AIS case 3) was a left MCA stroke complicated by a tPA hemorrhage. This case was previously published and tested in a simulation scenario with robust validity evidence, which allowed us to benchmark our residents' performance.^16^ AIS cases 1 and 2 are available in eAppendices 2 and 3.

### Competency Thresholds Determined by the Angoff Standard Setting Method

For the 2 AIS cases in which the residents were individually evaluated, the checklist of critical actions was developed by consensus. Once developed, we circulated the critical action list to a group of 12 multidisciplinary attendings (neurocritical attendings [7], vascular neurologists [4], and emergency medicine attendings [1]) at 6 different institutions. Each attending was asked to estimate the percentage of “minimally competent” and “well-prepared” residents that would complete each critical action. “Minimally competent” was defined as “a junior resident who is only borderline ready to become a senior resident and likely still requires some supervision.” “Well prepared” was defined as “a junior resident who is clearly ready to become a senior resident and could be entrusted to initiate appropriate care in many scenarios.” As per the Angoff method, the average of the minimally competent percentages was used to set the MPS for case 1 (right MCA stroke with blood pressure dependence) and case 3 (left MCA stroke with tPA hemorrhage); the average of the “well-prepared” percentages was used to set a Mastery Score (MS) for the same 2 cases. The MPS was set to 66% (13 of 20 checklist items) for case 1 and 61% (15 of 25 checklist items) for case 3. The MS was set at 85% (17 of 20 checklist items) for case 1 and 82% (20.5 of 25 checklist items) for case 3. All threshold scores were rounded to the closest 0.5 as there were 3 checklist items for which residents could receive a half point.

### Simulator and Simulation Environment

Through the prebrief, the faculty oriented the participants to the limitations of both the “live actor patient” and the manikin, as well as established a safe learning environment. Given that we wanted to fully evaluate how residents performed the NIHSS and the limitation of the manikin to perform many neurologic functions, in the scored AIS cases, participants interacted with a “live actor patient.” This live actor was a senior neurology resident, neurocritical care fellow, or neurocritical care attending—all of whom were coached in the standardized portrayal of a left or right MCA syndrome. Participants were instructed to collect information from the embedded “family member,” who was also a senior resident or fellow. The participants called consults as needed; the faculty member scoring the case answered the consults. Each case required a minimum of 3 course faculty—one to score the case, operate the vital signs, and provide confirmation of medication administration; one to act as the patient; and one to act as the family member.

The neurologic emergency simulation bootcamp took place in our center's simulation laboratory. For AIS case 1, the patient was portrayed by a senior resident or neurocritical care fellow. The room represented a typical emergency department room. Vitals included cycled blood pressures, continual pulse oximetry, and telemetry, which were displayed on a monitor. If requested, patient's radiologic images and chart data were displayed on the in-room television. Vitals were adjusted as the simulation progressed based on the intervention of the participant. When a medication was requested, an overhead voice would read back that the medication was through via intravenous push or continuous infusion, as appropriate.

For AIS case 2, which was the unscored case in which residents practiced AIS care, SimMan3G manikin (Laerdal Medical) was used. The simulation environment was the same as for AIS case 1. A fellow or senior resident played the role of a family member as an embedded participant.

**Figure 2:**
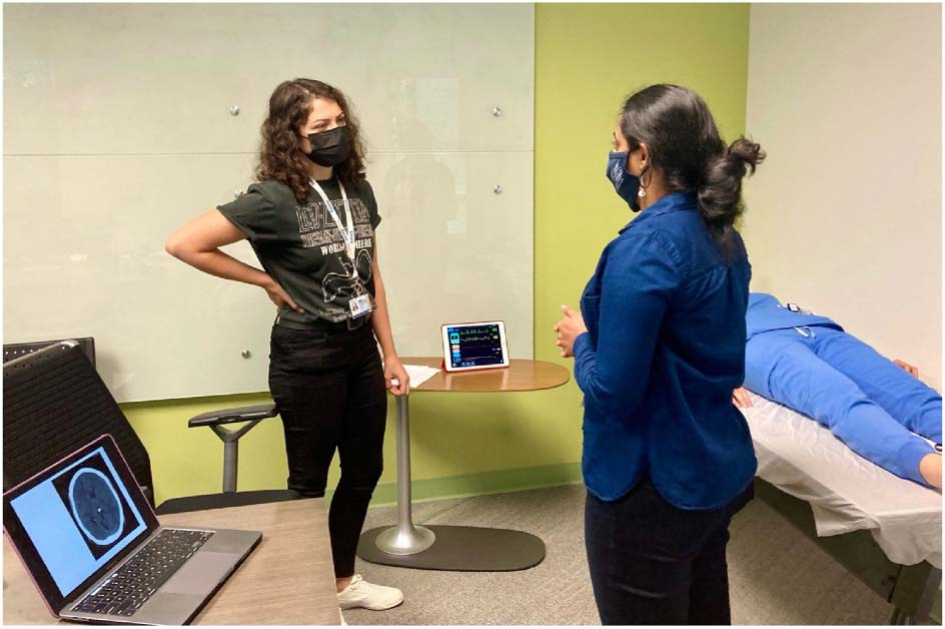
Simulation in the Mobile Sim Lab In the Mobile Sim Lab, vitals were displayed on an iPhone-controlled iPad and radiology was displayed on a laptop. There was no other medical equipment provided. Medications were “administered” by the simulation administer reporting that they had been given.

AIS case 3 was the unannounced, postcourse assessment test that took place during the first 8 weeks of the academic year (2–8 weeks after the initial simulation day, median 6 weeks). During the interval between pretest and posttest, 4 of the 13 junior residents participated in a stroke. Both junior and senior residents completed AIS case 3 during their protected didactic time. As such, the follow-up case was held in traditional classrooms, not in a simulation laboratory. We termed this the “Mobile Sim Labf” (Figure 2). The “bed” was a make-shift bed on a table, and the vitals were displayed on an iPad using *Simpl*—an app that syncs an iPad and iPhone. The iPhone was used to manipulate the vitals, and the iPad displayed the vitals in real time. There were no medications or airway equipment for when the patient would need to be “intubated.” The environment was a lower fidelity standard. Like in AIS case 1, the “patient” was still a live actor—a resident or fellow trained on which aspects of the NIHSS to portray.

### Clinical Scenarios

#### AIS Case 1

Residents cared for a patient with an NIHSS of 8 due to a right MCA M1 occlusion. The patient was hypertensive on arrival (systolic blood pressure >185 mm Hg) and had new onset atrial fibrillation. The family member reported a history of heavy alcohol use in the past and questioned intoxication as the reason for the apparent “confusion.” Once the physician recognized the acute stroke syndrome, they were to obtain last seen well (2 hours prior) and initiate a stroke code including activating the mechanical thrombectomy team. The full assessment checklist is presented in Table 2. Given the hypertension, trainees needed to lower blood pressure before tPA administration. This took several doses of IV push boluses or titrating a nicardipine infusion. After administering tPA, the patient's examination worsened. The resident was required to consider a differential for worsening, identify hypotension, and augment cerebral perfusion. Considering a tPA-related hemorrhage was appropriate if the low blood pressure was not recognized, but if the resident requested a repeat CT head, there was no intracranial hemorrhage.

**Table 2:**
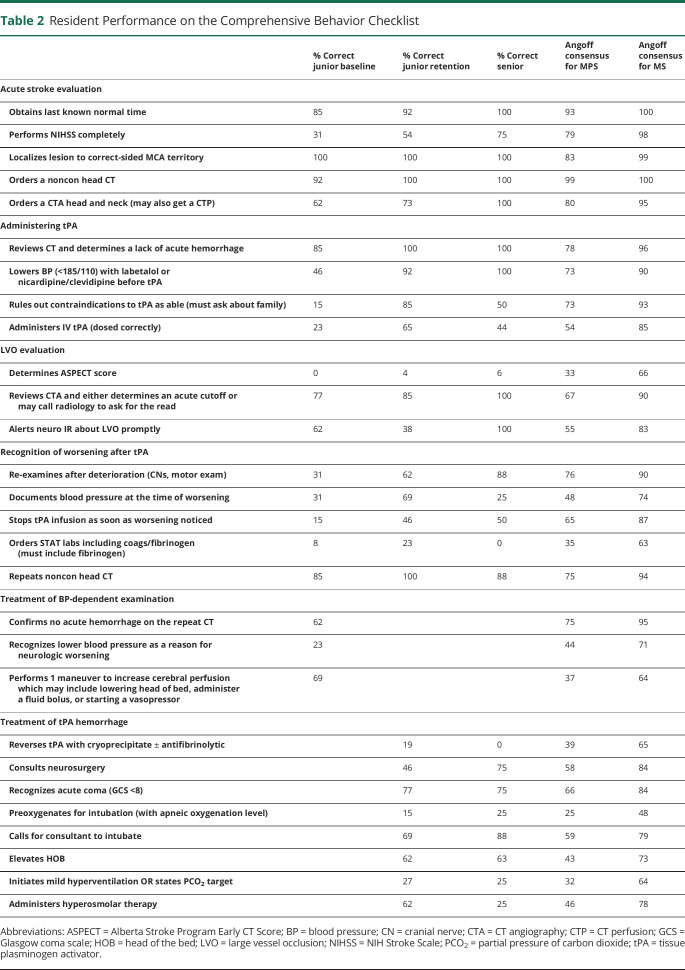
Resident Performance on the Comprehensive Behavior Checklist

	% Correct junior baseline	% Correct junior retention	% Correct senior	Angoff consensus for MPS	Angoff consensus for MS
Acute stroke evaluation					
Obtains last known normal time	85	92	100	93	100
Performs NIHSS completely	31	54	75	79	98
Localizes lesion to correct-sided MCA territory	100	100	100	83	99
Orders a noncon head CT	92	100	100	99	100
Orders a CTA head and neck (may also get a CTP)	62	73	100	80	95
Administering tPA					
Reviews CT and determines a lack of acute hemorrhage	85	100	100	78	96
Lowers BP (<185/110) with labetalol or nicardipine/clevidipine before tPA	46	92	100	73	90
Rules out contraindications to tPA as able (must ask about family)	15	85	50	73	93
Administers IV tPA (dosed correctly)	23	65	44	54	85
LVO evaluation					
Determines ASPECT score	0	4	6	33	66
Reviews CTA and either determines an acute cutoff or may call radiology to ask for the read	77	85	100	67	90
Alerts neuro IR about LVO promptly	62	38	100	55	83
Recognition of worsening after tPA					
Re-examines after deterioration (CNs, motor exam)	31	62	88	76	90
Documents blood pressure at the time of worsening	31	69	25	48	74
Stops tPA infusion as soon as worsening noticed	15	46	50	65	87
Orders STAT labs including coags/fibrinogen (must include fibrinogen)	8	23	0	35	63
Repeats noncon head CT	85	100	88	75	94
Treatment of BP-dependent examination					
Confirms no acute hemorrhage on the repeat CT	62			75	95
Recognizes lower blood pressure as a reason for neurologic worsening	23			44	71
Performs 1 maneuver to increase cerebral perfusion which may include lowering head of bed, administer a fluid bolus, or starting a vasopressor	69			37	64
Treatment of tPA hemorrhage					
Reverses tPA with cryoprecipitate ± antifibrinolytic		19	0	39	65
Consults neurosurgery		46	75	58	84
Recognizes acute coma (GCS <8)		77	75	66	84
Preoxygenates for intubation (with apneic oxygenation level)		15	25	25	48
Calls for consultant to intubate		69	88	59	79
Elevates HOB		62	63	43	73
Initiates mild hyperventilation OR states PCO_2_ target		27	25	32	64
Administers hyperosmolar therapy		62	25	46	78

Abbreviations: ASPECT = Alberta Stroke Program Early CT Score; BP = blood pressure; CN = cranial nerve; CTA = CT angiography; CTP = CT perfusion; GCS = Glasgow coma scale; HOB = head of the bed; LVO = large vessel occlusion; NIHSS = NIH Stroke Scale; PCO_2_ = partial pressure of carbon dioxide; tPA = tissue plasminogen activator.

#### AIS Case 2

Per the report, EMS had been called for concern for “jerking” and found the patient unresponsive at home. Residents were required to consider a differential diagnosis for acute onset altered mental status—including seizures, posterior circulation stroke, and toxic-metabolic causes. If trainees paused sedation, the examination revealed pinpoint pupils, ophthalmoparesis (displayed as a video clip of a real patient), and motor asymmetry—concerning for a brainstem process. CT of the head was normal. If they did not obtain a CT angiography (CTA), the embedded participant would make a comment about “what if blood is not getting to the brain” to prompt this scan. Once the clot was identified residents practiced safe administration and consent for tPA as well as alerting the endovascular team. During the team debrief, residents were encouraged to practice any aspect of stroke care they still felt uncertain about.

#### AIS Case 3

This case had many shared features with AIS case 1. However, when the patient worsened after the administration of tPA, it was due to a tPA-related hemorrhage. On identification of the intracranial hemorrhage, residents needed to stop the tPA infusion and initiate tPA reversal protocols. They were also expected to recognize a mental status change contributing to poor airway protection and recommend emergent endotracheal intubation. The case concluded when the patient was stabilized and recommended for NeuroICU admission.

### Outcomes

The primary outcome was the proportion of junior residents that achieved a MPS or a MS on the follow-up assessment (case 3). Secondary outcomes included the comparison of the junior resident performance to senior resident performance, changes in the percentage of correct answers on the multiple-choice knowledge test, changes in the confidence self-assessment, and the proportion of junior residents that achieved MS.

All simulations were viewed and graded by a single neurointensivist (C.S.W.A.) at the institution who was not blinded to the participants' PGY level or precourse/postcourse. Residents had to clearly demonstrate the full skill to receive a point, except for 3 skills where a half point was allowed. A half point was awarded if the resident ordered a CTA head and neck, but only after getting the result of the head CT; if the resident knew the dose of tPA was 0.9 mg/kg but did not know what percentage was to be administered as a bolus; or if the resident stopped tPA but only after discovery of ICH (the full point was awarded only if tPA was stopped when neurologic worsening occurred).

Residents also self-assessed their confidence in 12 stroke skills on a Likert scale ranging from “not at all confident” (score 1) to “extremely confident” (score 7). They also provided a confidence rating for the global “acute ischemic stroke” ENLS competency.

### Reliability

To assess the reliability of the assessment, an important component of validity 12 simulations (35%), which were randomly selected and represented a mix of junior precourse and postcourse assessments and PGY-3 simulations, were graded by a second neurointensivist (E.J.S.) at our institution. Any disagreements were resolved through discussion to reach a consensus.

### Anonymous Feedback and Simulation Fidelity Survey

All junior residents were asked to anonymously complete a 5-question, 5-point Likert scale survey about the simulation curriculum for enjoyment and course satisfaction. They also rated each case on a Likert scale for realism and level of difficulty and could provide open-ended feedback about ways to improve the course or what had been successful about the course. After completing the post-test in the Mobile Sim Lab, residents were sent a 3-question—“yes, no, maybe”—response format survey about the how their experience in the low-fidelity simulation environment compared with learning in the institutional simulation laboratory.

### Statistical Analysis

We reported descriptive statistics as mean (SD) for continuous variable and counts and frequencies for categorical variables. Using paired samples *t* tests, we compared junior resident simulation performance before and after the didactics plus simulation course, junior resident knowledge assessment before and after the didactics and didactics plus simulation course, and junior resident confidence before and after the didactics plus simulation course. Using independent samples *t* tests, we compared junior resident simulation performance postintervention with senior resident simulation performance, junior resident knowledge assessment postintervention with senior resident knowledge assessment, and junior resident confidence postintervention with senior resident confidence. We used a paired samples *t* test to compare simulation performance on 10 shared critical action items between the first simulation and follow-up simulation in junior residents. Cohen *d* was used to calculate effect size.^17^ We used the intraclass correlation (ICC) to assess agreement among raters with an ICC value of greater than 0.75 considered as excellent. The results were considered statistically significant if the *p* < 0.05. All analyses were performed using IBM SPSS Statistics 27. The reporting format is in accordance with the guidelines established by the Strengthening the Reporting of Observational Studies in Epidemiology study as well as the extended guidelines for health care simulation research.

### Standard Protocol Approvals, Registrations, and Patient Consents

This study was approved by the Emory University Institutional Review Board. All subjects signed e-consents to participate in the research curriculum and to be filmed during the simulation.

### Data Availability

On reasonable request, the data that support the findings of this study are available from the corresponding author (C.S.W.A).

## Results

Thirteen junior residents (9 PGY-2 adult neurology residents and 4 PGY-3 child neurology residents) participated in the course. Eight senior residents completed the follow-up simulation and served as the comparator group. Table 1 presents the baseline characteristics of the participating junior residents: Most reported very limited neurology exposure during their intern year and nearly half of the junior residents had never completed an NIHSS.

### Simulated Performance

**Figure 3:**
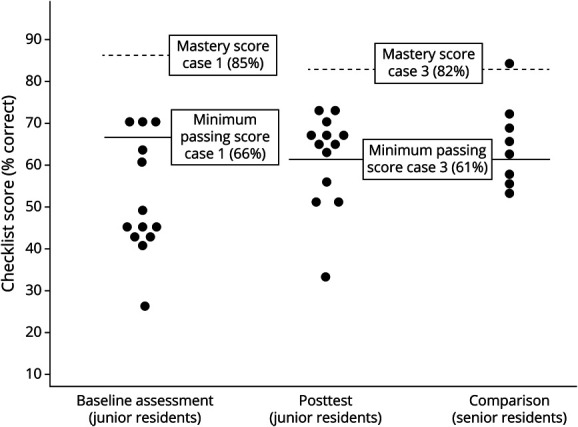
Residents Attaining Minimal Passing and Mastery Score Simulation performance score at baseline (AIS case 1) and post-test (AIS case 3) of 13 residents. Far right column demonstrates the performance of the PGY-3 comparators. The minimum passing score was set at 66% for case 1 and 61% for case 3. The Mastery Score was set to 85% for case 1 and 82% for case 3. Each circle represents an individual learner. AIS = acute ischemic stroke; PGY = postgraduate year.

All 13 residents completed the baseline simulation assessment (case 1) and the post-test (case 3). See Figure 3 for changes in MPS and MS. While only 3 residents (23%) achieved the MPS on the baseline assessment (case 1), 9 residents (69%) achieved the MPS on the post-test. For case 1, the average score on the performance checklist was 10.3 of 20 (53%) available points which significantly improved with a large effect size in case 3 to 62.8% (mean [SD] = 15.7 [2.6]) points of 25 available, *d* = 1.7, *p* < 0.001. On the 17 shared checklist items between case 1 and case 3, there was a large effect of the intervention on performance (case 1 mean score [SD] = 8.5 items [2.8] vs case 3 mean score [SD] = 11.9 [1.7], *d* = 1.4, *p* < 0.001). All but 1 trainee's performance in the shared elements improved between baseline and follow-up assessment.

There was no difference between postcourse performance of junior residents who had participated in their 2-week long stroke rotation and those who had not (junior residents with stroke rotation score mean [SD] = 15.1 [2.5] vs junior residents without stroke rotation mean [SD] = 15.9 [2.9], *p* = 0.658). Of the 4 junior residents with clinical stroke experience before their postcourse assessment, 2 achieved the MPS and 2 did not.

Eight senior residents completed case 3, 5 (63%) reached the MPS, and 1 achieved the MS. Senior residents' average score on the performance checklist was 64% (mean score [SD] = 16 [2.5]). There was no difference between simulation scores of the postcourse juniors and senior residents (postcourse junior resident simulation mean score [SD] = 15.7 [2.6] vs senior resident simulation mean score [SD] = 16.0 [2.5], *p* = 0.793).

### Change in Knowledge

Twelve residents completed the precourse, predidactics knowledge assessment; 11 completed the precourse postdidactic knowledge assessment; and 12 completed the postcourse, postdidactic knowledge assessment. Predidactic, presimulation course, the mean score on the 20 knowledge questions assessing acute stroke management was 40% (mean number correct [SD] = 7.8 [1.9]). After the “top 10 pearls” didactics, the mean score rose to 52.2% (mean number correct [SD] = 10.5 [1.9]). After the simulation course, the mean knowledge score rose to 56.3% (mean number correct [SD] = 11.3 [2.3]). The change from precourse to postcourse was significant (*p* < 0.001), with a large effect size (*d* = 1.3). The 9 senior residents average score on the knowledge assessment was 60.5% (mean number questions correct [SD] = 12.1 [2.5]). There was no significant difference between postcourse junior residents' scores (mean correct [SD] = 11.3 [2.3]) and senior residents' scores (mean correct [SD] = 12.1 [2.5]), *p* = 0.421.

### Change in Confidence

**Figure 4:**
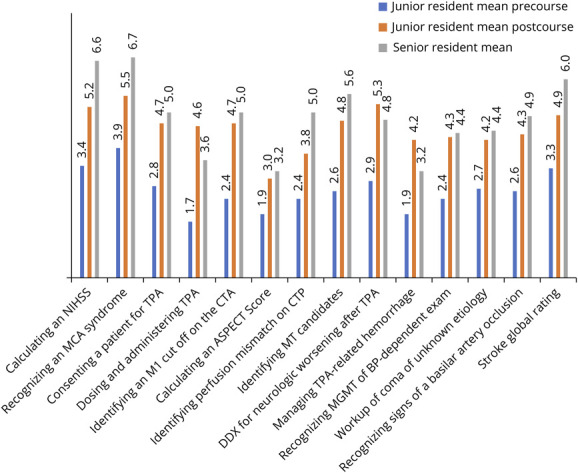
Self-Reported Mean Confidence Scores for Acute Ischemic Stroke Skills Junior and senior residents rated their confidence in subcategories of acute ischemic stroke management on a 1–7 Likert scale with 1 representing “not at all confident” and 7 representing “extremely confident.” They also rated their confidence on AIS globally, which is depicted on the far right column “stroke global rating.” ASPECT = Alberta Stroke Program Early CT Score; BP = blood pressure; CTA = CT angiography; CTP = CT perfusion; DDX = differential diagnosis; MCA = middle cerebral artery; MGMT = management; MT = mechanical thrombectomy; NIHSS = NIH Stroke Scale; tPA = tissue plasminogen activator.

The mean self-reported confidence for AIS skills among all groups (precourse juniors, postcourse juniors, and seniors) is presented in Figure 4. Mean global confidence rating for AIS increased after the course with a large effect size (mean self-reported confidence score precourse [SD] = 3.3 [1.9] vs mean self-reported confidence score postcourse [SD] = 4.9 [1.2], *d* = 1.4, *p* < 0.001). There was no difference in reported confidence between junior residents' postintervention and senior residents (junior resident self-reported confidence postintervention mean [SD] = 4.9 [1.2] vs senior resident self-reported confidence mean [SD] = 5.7 [1.0], *p* = 0.154).

### Inter-rater Reliability

Inter-rater reliability was excellent for the sum score of critical action items (ICC = 0.96; *p* < 0.001). One area for clarification in the scoring was if residents were required to specify the side of the occlusion during the initial evaluation. In adjudication we determined that residents should receive a point for knowing the correct side if they identified the syndrome (i.e., said, “this is a left/right MCA syndrome”) at any point during the simulation. Other discrepancies in the scores clustered around if the residents had completed a comprehensive NIHSS score (e.g., could you assume that the resident had identified aphasia if they did not specifically use the NIHSS stroke cards?) and thorough consent for thrombolysis administration. We allowed the scoring differences to persist without resolution as they made little impact in the overall assessment of the resident's performance.

### Anonymous Feedback

Seven residents provided feedback on the Likert scale questions. All residents reported that they learned a great deal and enjoyed the simulation curriculum. Six residents (89%) agreed they preferred doing simulation to traditional didactics, felt the curriculum lessened their anxiety about responding to neurologic emergencies, and would be interested in doing simulation in the future. “Strongly agree” was the majority response (68%) for all questions regarding “was the case realistic?” and “was the case the right level of difficulty?”

### Acceptance of “Low-Fidelity Sim Lab”

Eleven residents provided an evaluation of the low-fidelity simulation environment (“The Mobile Sim Lab”). When asked if having the simulation in “The Mobile Sim Lab” affected their ability to engage with the simulation, 9 residents (82%) reported that it did not, 1 reported “somewhat,” and the other was “yes.” When asked if the “Mobile Sim Lab” affected how much they learned, 9 again reported “no” and the other 2 votes were for “yes” or “maybe.” Comments included “I focused on the case and having a live actor was still helpful,” “… I think it was easier ‘to come out of character’ with the mobile sim experience, but truthfully it did not affect my experience.”

## Discussion

Using the Angoff standard setting method to determine a MPS for AIS management, we found that with just 1 simulation bootcamp most junior residents could attain a MPS for AIS care, but that the intervention did not result in mastery. In addition, after the AIS simulation course, junior residents performed similar to senior residents in the simulated management of AIS with tPA-related hemorrhagic conversion (case 3). Through a detailed item checklist, we were able to distill which performance metrics improved after a simulation course, thus documenting a behavioral change—a high-level achievement in the Kirkpatrick model of curriculum evaluation.^18^

As expected, junior residents had limited knowledge and confidence before the curriculum. While trainees' knowledge score significantly improved after didactics, only 3 residents achieved the MPS and none achieved the MS on the baseline assessment. The mean checklist score was low with residents achieving on average 10.3 of 20 possible points (53%). This baseline performance score mirrors those in other assessments of junior residents in neurologic emergencies. For example, in the SBML curriculum for SE, no resident attained the MPS in the pretest and the mean checklist score was less than 50%.^10^ Similarly, in another AIS study, “level 2” residents (which included PGY-2 residents) successfully completed on average 56% of the task checklist in a case that shared many similar features.^16^

Having traditional didactics before the simulation course, a postdidactic multiple-choice assessment allowed us to measure what knowledge residents gained from didactics and how that knowledge translated to clinical performance. For example, on the postdidactic multiple-choice assessment, 100% of residents selected the correct blood pressure goals for tPA administration, but in the baseline simulation assessment, less than half of trainees lowered blood pressure at all or to the correct threshold. Similar findings were seen with high knowledge scores and low performance scores in correct dosing for alteplase (written assessment = 81% correct and simulated assessment = 23% correct) and ability to calculate an Alberta Stroke Program Early CT Score (written assessment = 55% correct and simulated assessment = 0% correct). These findings help illustrate why in the Kirkpatrick model of curriculum assessment, assessing for a behavioral change—that is, that the learner can translate their knowledge into action—is a higher level for evaluating a curriculum's effectiveness than a written knowledge assessment.^17^ There are likely many reasons why written knowledge does not always translate to performance. One important difference is that in simulation (and clinical practice), there are competing priorities and stressors. Simulation not only assesses the trainees' knowledge but also valuable skills of triage, communication, and leadership—what are termed “nontechnical” skills in the Crisis Resource Management literature.^19^ It is crucial that trainees develop these skills as they are critical for high-performing acute stroke teams.^20^

Residents in our study significantly improved on the simulation post-test with 69% achieving the MPS. However, despite the simulation course, none achieved mastery. Our results contrasts those from SBML courses in which all learners achieved mastery on the post-test and in either in situ evaluation or clinical performance.^10,21,22^ This is likely due to the mastery learning (ML) emphasis^9^: When a standard simulation curriculum and ML curricula were compared, students in the ML curriculum had superior performance at 1 year.^23^ While ML curricula can promote sustained excellence, they are often time and resources intensive. For example, in the SE course, nearly half of residents did not pass the first post-test and required further deliberate practice; the average time to the final post-test was 71.25 days. By contrast, standard simulation bootcamps require less time investment and are feasible for many programs; this learning paradigm has already been embraced by many neurology training programs.^3–8^ The findings in this study confirm what has been documented in other specialties that simulation bootcamps can accelerate competency among novice learners.^24–26^

Although there was a small sample size of senior residents, their performance in the simulation mirrors what has previously been reported: Senior residents who have clinical experience in caring for stroke patients perform well on the basics of stroke care.^16^ Seniors were generally adroit at expediting the workup to the CT scanner, performing an NIHSS, lowering blood pressure, and alerting the endovascular team to a large vessel occlusion. However, like what was found by Pergakis et al., our trainees performed poorly in the management of tPA-related hemorrhagic conversion—a low-frequency, high-acuity scenario. This provides further generalizability evidence that standard clinical training may be inadequate preparation for low-frequency scenarios in acute stroke care and other neurologic emergencies. For example, in a recent study of residents, more than half failed to consider viral meningitis as an etiology of SE, another low-frequency, high-acuity event,^27^ and “ready to graduate” senior residents performed poorly on a simulation assessment of SE.^28^ That standard clinical training may fail to result in mastery among senior neurology residents has also been demonstrated in the domain of procedural training: In a study of senior neurology residents performing lumbar punctures, only 6% met a MPS set by an expert panel.^21^ It is thus not unexpected that we discovered areas for improvement among our senior trainees.

Although not the primary aim of our study, the situation dictated that we conduct the follow-up simulations in a low-fidelity environment, which we called “The Mobile Sim Lab.” After the post-test, residents were queried on whether the lower fidelity environment affected their ability to suspend disbelief and engage in the simulation. For the large majority, it did not. Similarly, most residents reported that it did not affect how much they learned from the simulation. These findings are subject to bias and will need to be explored further in studies. However, this was an important take away for our experience. One of the most difficult elements of implementation was scheduling time in the simulation laboratory, which is remote from our resident's didactics and clinical rotations. Assessing that our residents had a near equivalent experience in the Mobile Sim Lab was crucial for future curriculum planning. More generally, this is an important finding as a commonly cited limitation to the implementation of simulation is access to a high-fidelity training environment.^29^ Our experience provides further evidence that the level of fidelity may be of secondary importance to elements that can be accomplished in all simulations: deliberate practice in a safe learning environment and structured debriefing.^30–33^

This study has several limitations. There is a small sample size. Given this was conducted at a single institution, we did not calculate a sample size, but strived to enroll all junior and PGY-3 residents; we achieved 96% enrollment. It was a single-center experience and may also limit generalizability. However, the assessment case was previously studied at a separate institution with similar findings which does suggest that the performance results are more widely generalizable. As residents were assessed up to 8 weeks after the simulation course, improvement in the simulation may also be attributable to skills learned in clinical care; however, on average, the residents that had a stroke rotation between the preassessment and postassessment performed the same as those that did not. The size of this group is too small to draw any conclusions as to why the performance improvement was not more striking. Notably, there was a wide range of post-test scores for this group (range 13–17.5). Two did perform substantially better on the simulation post-test than on their pretest, and their improvement may have biased the favorable trend seen overall.

Neither rater was blinded to the PGY of the participant, and they were both from the same institution as the participants. However, the checklist items were binary, and we found excellent inter-rater reliability among the randomly selected group of precourse and postcourse assessments that was reviewed by a second rater. Although all the seniors received prebriefing about the limitations and expectations of the simulation experience, they lacked previous simulation experience. Thus, perhaps they missed points that they would have performed in the care of a real stroke patient because they were unfamiliar with the environment. Similarly, junior participant assessment in a low-fidelity environment may have affected their performance, even if few felt that there was any difference. It is possible that if residents performed the post-test assessment in a higher fidelity environment or with a real patient, more would have obtained the MPS or the MS. This study did not capture why trainees made the mistakes they did, even if these frames were explored in the debriefing. Understanding cognitive frameworks and failed heuristics of novice trainees should be explored in future studies. Another possible limitation is that the Angoff method relies on expert consensus. We surveyed a broad range of attendings across multiple institutions and subspecialties that have experience in caring for acute stroke patients. However, there is no validity evidence to support that our cutoffs for a MPS and MS are the “gold-standard” thresholds. In addition, when assessing knowledge, trainees took the same multiple-choice test 3 times. Although no correct answers were provided until the final test, residents may have improved on the test just because of their familiarity with the questions.

Finally, it will be important to demonstrate a translational effect of this training. Future work will need to appraise if the behavior change measured in the simulation laboratory translated into reduced door-to-needle or door-to-groin times in clinical practice.

A 1-day simulation bootcamp resulted in significant improvement in the number of residents achieving a MPS. Although none achieved mastery on the post-test, their performance was comparable with senior residents. Learners also felt more confident and demonstrated higher knowledge on the postcourse multiple choice assessment. Further study is needed to understand how these skills would translate to clinical performance in the hospital setting.

## Appendix Authors

**Table:**

Name	Location	Contribution
Catherine S.W. Albin, MD	Department of Neurology and Neurosurgery, Emory University School of Medicine, Atlanta, GA	Drafting/revision of the manuscript for content, including medical writing for content; major role in the acquisition of data; study concept or design; analysis or interpretation of data
Melissa B. Pergakis, MD	Department of Neurology, Program in Trauma, University of Maryland School of Medicine, Baltimore	Drafting/revision of the manuscript for content, including medical writing for content; study concept or design; analysis or interpretation of data
Erika J. Sigman, MD	Department of Neurology and Neurosurgery, Emory University School of Medicine, Atlanta, GA	Drafting/revision of the manuscript for content, including medical writing for content; major role in the acquisition of data; analysis or interpretation of data
Nirav R. Bhatt, MD	Department of Neurology, University of Pittsburgh School of Medicine, PA	Major role in the acquisition of data; study concept or design
Spencer K. Hutto, MD	Department of Neurology, Emory University School of Medicine, Atlanta, GA	Major role in the acquisition of data; study concept or design
Sitara Koneru, MD	Department of Neurology, Emory University School of Medicine, Atlanta, GA	Major role in the acquisition of data; study concept or design
Ehizele M. Osehobo, MD	Department of Neurology, Emory University School of Medicine, Atlanta, GA	Major role in the acquisition of data; study concept or design
Joaquin A. Vizcarra, MD	Department of Neurology, Emory University School of Medicine, Atlanta, GA	Drafting/revision of the manuscript for content, including medical writing for content; major role in the acquisition of data; study concept or design
Nicholas A. Morris, MD	Department of Neurology, Program in Trauma, University of Maryland School of Medicine, Baltimore	Drafting/revision of the manuscript for content, including medical writing for content; major role in the acquisition of data; study concept or design; analysis or interpretation of data

## Glossary

AIS: acute ischemic stroke
CTA: CT angiography
ENLS: Emergency Neurological Life Support
ICC: intraclass correlation
ICH: intracerebral hemorrhage
MCA: middle cerebral artery
ML: mastery learning
MPS: Minimum Passing Score
MS: Mastery Score
NIHSS: NIH Stroke Scale
PGY: postgraduate year
SBML: simulation-based mastery learning
SE: status epilepticus
tPA: tissue plasminogen activator
